# Cinobufagin Modulates Human Innate Immune Responses and Triggers Antibacterial Activity

**DOI:** 10.1371/journal.pone.0160734

**Published:** 2016-08-16

**Authors:** Shanshan Xie, Laura Spelmink, Mario Codemo, Karthik Subramanian, Katrin Pütsep, Birgitta Henriques-Normark, Marie Olliver

**Affiliations:** 1 Department of Microbiology, Tumor and Cell Biology, Karolinska Institutet, SE-171 77 Stockholm, Sweden; 2 Department of Clinical Veterinary Medicine, College of Veterinary Medicine, Jilin University, Changchun, Jilin, China; 3 Department of Clinical Microbiology, Karolinska University Hospital, SE-171 76 Stockholm, Sweden; University of Kansas Medical Center, UNITED STATES

## Abstract

The traditional Chinese medicine Chan-Su is widely used for treatment of cancer and cardiovascular diseases, but also as a remedy for infections such as furunculosis, tonsillitis and acute pharyngitis. The clinical use of Chan-Su suggests that it has anti-infective effects, however, the mechanism of action is incompletely understood. In particular, the effect on the human immune system is poorly defined. Here, we describe previously unrecognized immunomodulatory activities of cinobufagin (CBG), a major bioactive component of Chan-Su. Using human monocyte-derived dendritic cells (DCs), we show that LPS-induced maturation and production of a number of cytokines was potently inhibited by CBG, which also had a pro-apoptotic effect, associated with activation of caspase-3. Interestingly, CBG triggered caspase-1 activation and significantly enhanced IL-1β production in LPS-stimulated cells. Finally, we demonstrate that CBG upregulates gene expression of the antimicrobial peptides (AMPs) hBD-2 and hBD-3 in DCs, and induces secretion of HNP1-3 and hCAP-18/LL-37 from neutrophils, potentiating neutrophil antibacterial activity. Taken together, our data indicate that CBG modulates the inflammatory phenotype of DCs in response to LPS, and triggers an antibacterial innate immune response, thus proposing possible mechanisms for the clinical effects of Chan-Su in anti-infective therapy.

## Introduction

Chan-Su is a traditional Chinese medicine prepared from the dried secretion of the skin and parotid glands of the Chinese toad *Bufo bufo gargarizans*. It has been used for centuries as an Oriental drug for treatment of arrhythmia and other heart diseases, and the principle active components of Chan-Su include a group of bufadienolides which are responsible for its cardiotonic effects [1]. Chan-Su was introduced in Europe in the 17^th^ century, but was replaced by digitalis some 200 years later [2]. Chan-Su is also widely used to treat different types of cancers in China [3, 4] and, when used in combination with chemotherapy or radiotherapy, may enhance the efficacy of conventional therapies [5]. Several studies have shown that Chan-Su extracts and the active component cinobufagin (CBG) suppress cell proliferation and cause apoptosis of cancer cells *in vitro* via a sequence of apoptotic modulators, including caspases [6–9].

In addition to heart diseases and cancer, Chan-Su is also commonly used as a remedy for infections such as tonsillitis and acute pharyngitis [10]. However, to our knowledge, no antimicrobial compound has been isolated, and the capacity of Chan-Su extracts to kill bacteria *in vitro* is unknown. We thus hypothesized that Chan-Su extracts and/or CBG may either kill bacteria directly, or boost innate immune cell antibacterial activities.

Dendritic cells (DCs) represent a heterogeneous population of professional antigen-presenting cells which are involved in the initiation of inflammation in response to bacteria. They act as a major link between the innate and adaptive immune system [11], and are widely distributed in tissues throughout the body where they can interact directly with bacteria on mucosal surfaces [12]. The interaction of DCs with microorganisms triggers a cascade of pro-inflammatory cytokines which together orchestrate the early host response, and also shape the subsequent adaptive immunity. The pro-inflammatory cytokine interleukin-1 beta (IL-1β) plays an important role in the immune response to microorganisms. Children that are deficient in interleukin-1 receptor-associated kinase 4 (IRAK-4), which is involved in IL-1 signal transduction, have a selective predisposition to bacterial infections [13], and the absence of interleukin-1 receptor, type I (IL-1R1) or Myeloid differentiation primary response gene 88 (MyD88) increases morbidity and mortality from bacterial infections [14, 15]. Processing and release of IL-1β is primarily regulated by caspase-1 [16], which is regarded as a key mediator of inflammatory processes, and is controlled by an intracellular multi-protein complex termed the inflammasome [17].

Antimicrobial peptides (AMPs), including defensins and cathelicidins, are important components of the innate immune system, protecting the host from pathogens [18]. The highest concentrations of AMPs are found in tissues exposed to microbes, and in cells that are involved in host defense, such as monocytes/macrophages, DCs and neutrophils. Human neutrophils express AMPs that display microbicidal activities against a wide range of bacteria, fungi and viruses [19]. Besides being natural antibiotics in infected tissues, at lower concentrations these small cationic peptides can also act as signaling regulators to activate the immune system [20, 21]. In humans, there are two main defensin subfamilies, α-defensins (the intestinal Paneth cell defensins HD5 and HD6, and the human neutrophil peptides, HNP1-4) and β-defensins (hBD1-4) [22]. It has been demonstrated that intestinal epithelial cell secretion of the α-defensin HNP-1 can be triggered by the muscarinic receptor agonist carbachol [23, 24] but also by cytokines such as IL-13 [25]. Some AMPs, e.g. the α-defensins HNP1-3 and the cathelicidin LL-37, are chemotactic for monocytes, immature DCs and naïve T cells [26, 27], underpinning their immunoregulatory functions [27]. Human β-defensin 1 (hBD-1) is constitutively expressed whereas the expression of hBD-2 and hBD-3 are inducible by bacterial products [28, 29]. In addition to bacterial components, endogenous inflammatory mediators such as TNF-α, IL-1β and histamine can act as stimulants, enhancing the production of hBD-2 [30, 31].

The aim of this study was to explore the immunomodulatory properties of CBG, and we report for the first time that CBG modulates important immune functions such as DC maturation and cytokine production, and triggers the expression of AMPs in DCs and promotes the release of AMPs by neutrophils.

## Results

### Chan-Su and CBG do not have direct antimicrobial effects

First, we investigated whether Chan-Su had any direct *in vitro* antimicrobial activity against different bacterial species, and found that it was only bactericidal at very high concentrations. A 50% reduction in viable bacteria was observed after incubation with Chan-Su at a concentration of 15 mg/ml (*E*. *coli*), 5 mg/ml (*S*. *pyogenes*), or 1.5 mg/ml (*S*. *pneumoniae*), whereas complete killing of *S*. *pneumoniae* and *S*. *pyogenes* was observed at 15 mg/ml (data not shown). The serum concentration of Chan-Su is approximately 1 μg/ml in an individual who has ingested a moderate to large amount of Chan-Su [32], while the observed concentration required for antimicrobial activity was 10,000 fold higher. Consequently, we could rule out a direct antimicrobial effect of Chan-Su as its mechanism of action. Similarly, the active component CBG, at a concentration of 100 μg/ml, had no antimicrobial effect against these bacteria (data not shown). Thus, we hypothesized that Chan-Su might exert its anti-infective effects through modulation of innate immune responses, and henceforth focused our investigations on the effects of the active component CBG, on human DCs. We used mass spectrometry analysis to ascertain the purity of the CBG preparation from the Chinese National Institute for Food and Drug Control, and used another commercially available CBG as a reference and found that they disclosed similar mass spectra (see Material and Methods, S1 Fig). Furthermore, no detectable LPS contamination could be found as determined by the Limulus amebocyte lysate (LAL) test (S2 Fig).

### CBG inhibits DC maturation and cytokine production in response to LPS

**Fig 1:**
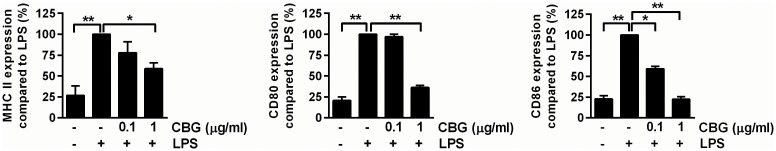
CBG inhibits DC maturation in response to LPS stimulation. DCs were stimulated with LPS (100 ng/ml) in the presence of vehicle or CBG for 24 hours. Cells were stained with anti-MHC class II, anti-CD80 and anti-CD86 antibodies and analyzed by flow cytometry. Data shown represent means + SEM of the Median Fluorescence Intensity (MFI), expressed as % compared to LPS alone, for 3 donors. *P<0.05.

The co-stimulatory molecules CD80 and CD86 are cell surface glycoproteins expressed on a variety of antigen presenting cells such as DCs. Coordinated up-regulation of CD80 and CD86, and translocation of major histocompatibility complex (MHC) to the cell surface are essential for DC maturation and antigen presentation to T cells. To examine the effect of CBG on DC maturation, we monitored surface expression of CD80, CD86 and MHC class II after 24 hours of stimulation with CBG and lipopolysaccharide (LPS) from *E*. *coli*, a known activator of DC maturation [33]. LPS-induced DC maturation was significantly inhibited by 1 μg/ml CBG (Fig 1) while stimulation of DCs with CBG alone had no effect on DC surface markers (data not shown).

**Fig 2:**
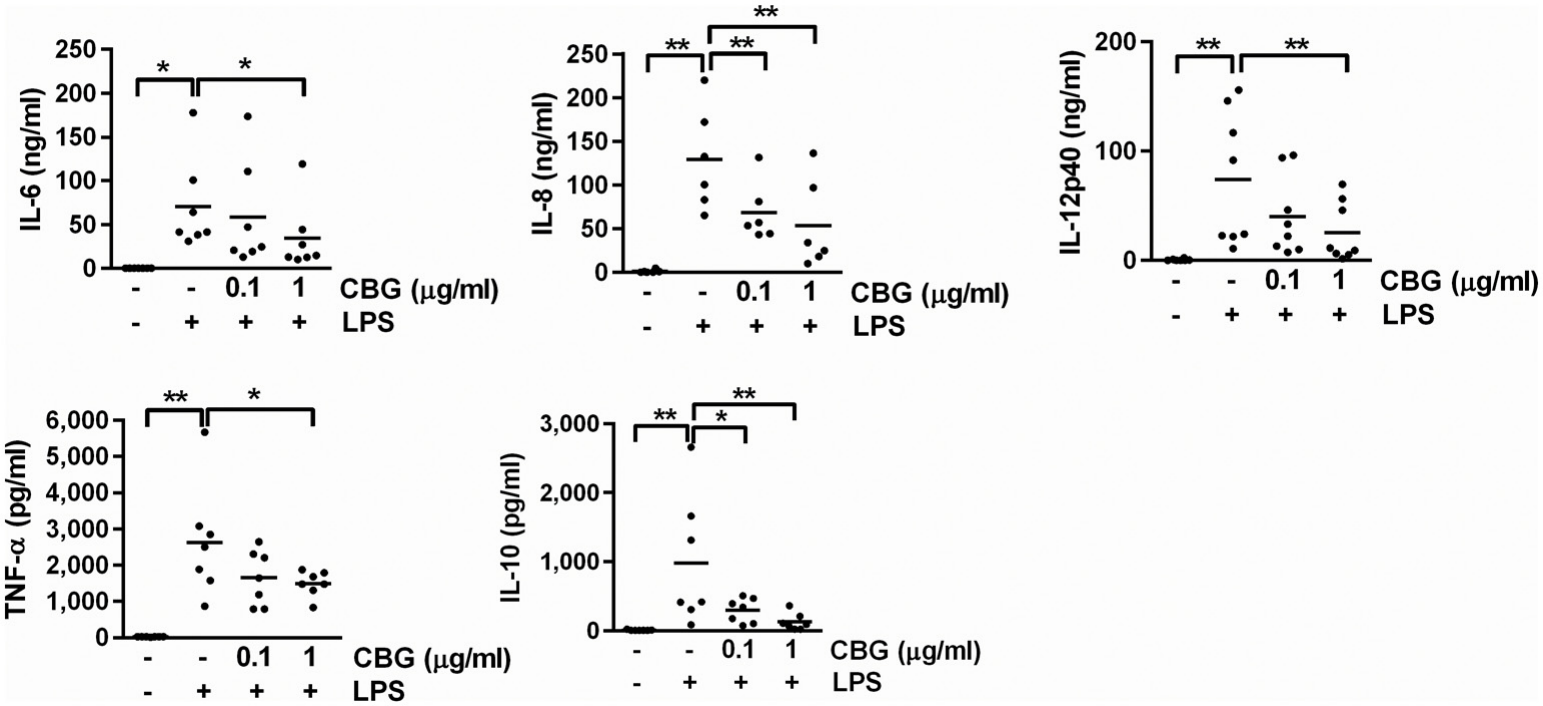
Inhibited cytokine release of LPS-stimulated DCs. DCs were stimulated with LPS (100 ng/ml) in the presence of vehicle or CBG for 24 hours. Supernatants were collected and analyzed for IL-6, IL-8, IL-12p40, TNF-α and IL-10. Data shown represent cytokine production for each donor and mean values for 6–8 donors. *P<0.05. **P<0.01.

A major element of DC activation, in addition to upregulation of co-stimulatory molecules, is release of cytokines and chemokines. To determine whether CBG can modulate DC production of cytokines and chemokines in response to LPS, we measured the levels of tumor necrosis factor (TNF)-α, interleukin (IL)-6, IL-8, IL-10 and IL-12p40 in supernatants of DCs following stimulation with LPS and CBG. LPS alone potently increased the production of all cytokines measured, whereas addition of CBG led to a strong inhibition of IL-6, IL-8, IL-10 and IL-12p40 (Fig 2). The cytokine response of DCs stimulated with CBG alone was similar to treatment with vehicle (S3 Fig).

### CBG exerts pro-apoptotic effects associated with caspase activation

**Fig 3:**
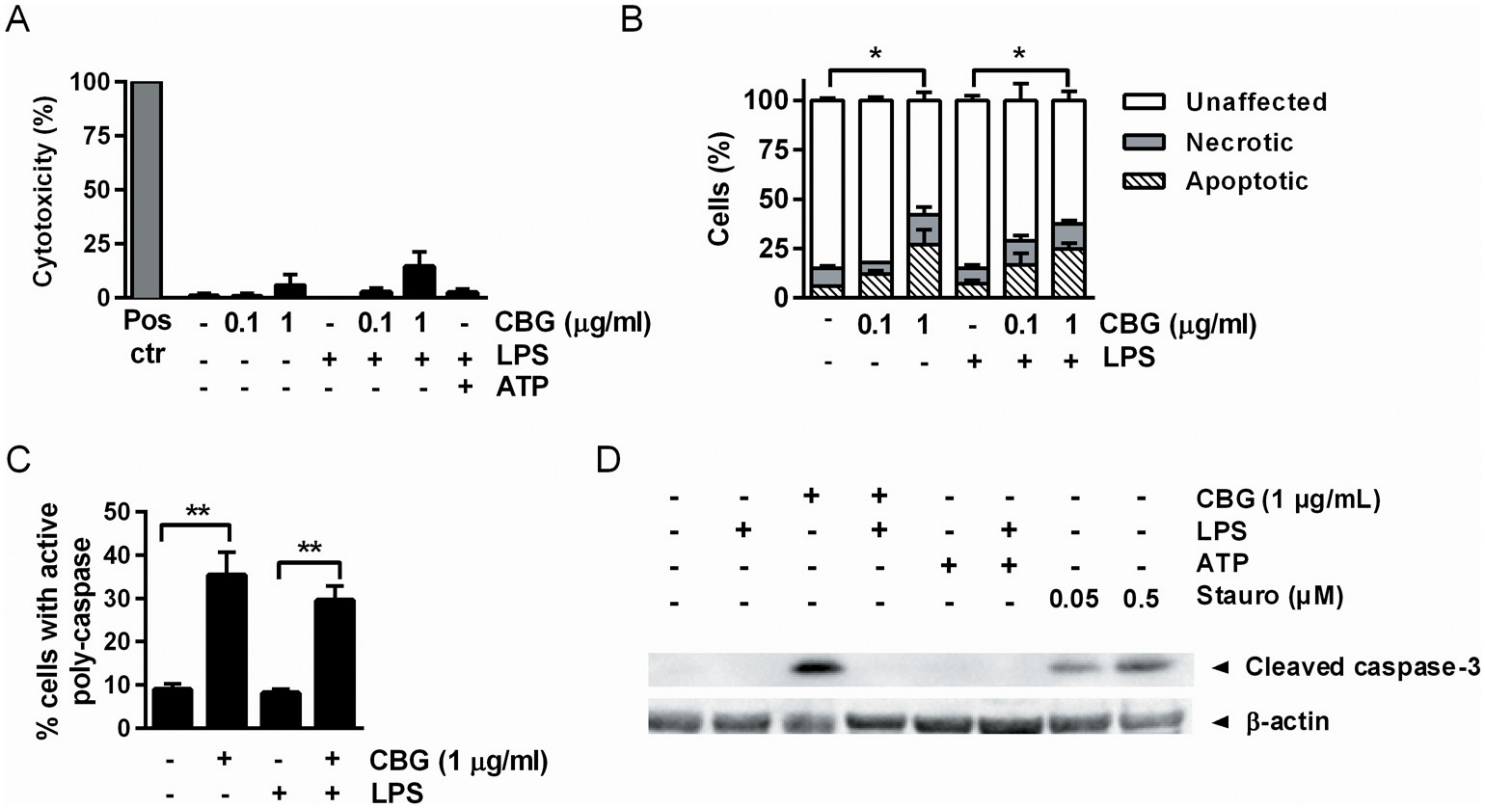
CBG exerts pro-apoptotic effects associated with caspase activation. DCs were stimulated with vehicle or CBG in the absence or presence of LPS (100 ng/ml) for 24 hours. (A) Supernatants were collected and analyzed for lactate dehydrogenase (LDH) release. Data shown represent means + SEM for 4 donors. Pos ctr, positive control (lysis with detergent; 100%). (B) Cell apoptosis and necrosis was examined by analyzing the percentage of Annexin V^+^ or Annexin V^+^ and PI^+^ cells, respectively. Data shown represent means + standard deviation (SD) for 1 donor, and are representative of the results of 3 donors. (C) Active caspases were detected with the FAM FLICA Poly Caspase Assay Kit, and data shown represent means + SEM for 4 donors. (D) Whole-cell extracts were prepared, and equal amounts of lysates were analyzed by Western blot using an antibody against caspase-3. β-actin was used as a loading control. The results shown are representative of three independent experiments. *P<0.05. **P<0.01.

Next the effect of CBG on DC viability was evaluated using the lactate dehydrogenase (LDH) assay. Stimulation with CBG at 1 μg/ml, either alone or combined with LPS, did not induce significant cytotoxicity (Fig 3A). To further examine the impact of CBG on cell viability, Annexin V was used in conjunction with propidium iodide (PI) to differentiate viable, apoptotic and necrotic cells. The number of apoptotic cells (Annexin V^+^PI^-^) was markedly increased by the addition of 1 μg/ml CBG, especially in the absence of LPS (Fig 3B). No significant increase in cell necrosis could be observed (Fig 3B), consistent with the LDH assay.

We also measured the activation of caspases in DCs, and found that exposure to CBG significantly increased the number of poly-caspase positive cells, both in the presence and absence of LPS (Fig 3C). Interestingly, addition of CBG alone induced activation of caspase-3, at levels similar to staurosporine which served as a positive control, however, this effect was lost when cells were co-stimulated with LPS (Fig 3D). It is possible that LPS activation extends the lifespan of the DCs as it has been shown previously that LPS may prevent apoptotic death of DCs [34].

### CBG triggers IL-1β release and caspase-1 activation

**Fig 4:**
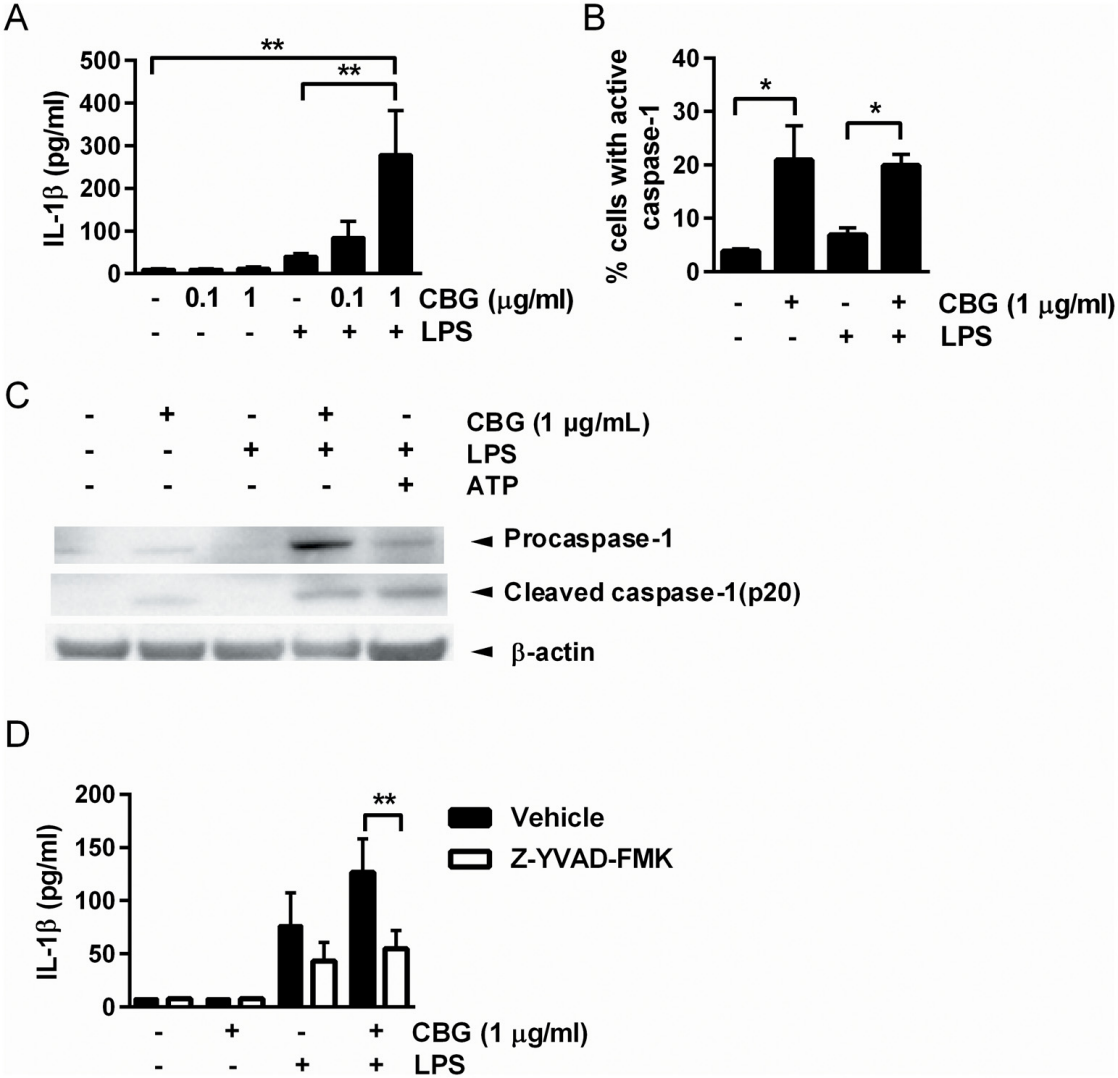
CBG triggers IL-1β production and caspase-1 activation. DCs were stimulated with vehicle or CBG in the absence or presence of LPS (100 ng/ml) for 24 hours. (A) Supernatants were collected and analyzed for IL-1β production. Data shown represent means + SEM of IL-1β production for 17 donors. (B) Inflammasome activation was examined by analyzing the percentage of caspase-1 positive cells. Data shown represent means + SEM for 3 donors. (C) Whole-cell extracts were prepared, and equal amounts of lysates were analyzed by Western blot using an antibody against caspase-1. β-actin was used as a loading control. The results shown are representative of three independent experiments. (D) Cells were pretreated with the caspase-1 inhibitor Z-YVAD-FMK for 1 hour. Supernatants were collected at 24 hours and analyzed for IL-1β production. Data shown represent means + SEM of IL-1β production for 3 donors. **P<0.01. *P<0.05.

In contrast to the inhibitory effect on various DC cytokines shown (Fig 2), CBG significantly upregulated production of the pro-inflammatory cytokine IL-1β in LPS-stimulated cells (Fig 4A). To further examine the effects of CBG on IL-1β production, we measured the expression of caspase-1, which is the protease responsible for the processing of pro-IL-1β to the active, secreted molecule. Flow cytometric evaluation of cells that had been stained with FAM-FLICA^™^ Caspase 1 demonstrated an increase in active caspase-1 in DCs stimulated with 1 μg/ml CBG in the presence or absence of LPS (Fig 4B). The presence of the active form of caspase-1 was also confirmed by Western blot analysis (Fig 4C). Moreover, addition of the caspase-1 inhibitor Z-YVAD-FMK markedly dampened secretion of IL-1β into the medium (Fig 4D). These results suggest that CBG has dual effects on the regulation of inflammatory processes, by dampening the production of multiple cytokines involved in inflammatory responses to LPS, and simultaneously enhancing capase-1-dependent processing and release of IL-1β.

### CBG upregulates the expression of AMPs in DCs and neutrophils

**Fig 5:**
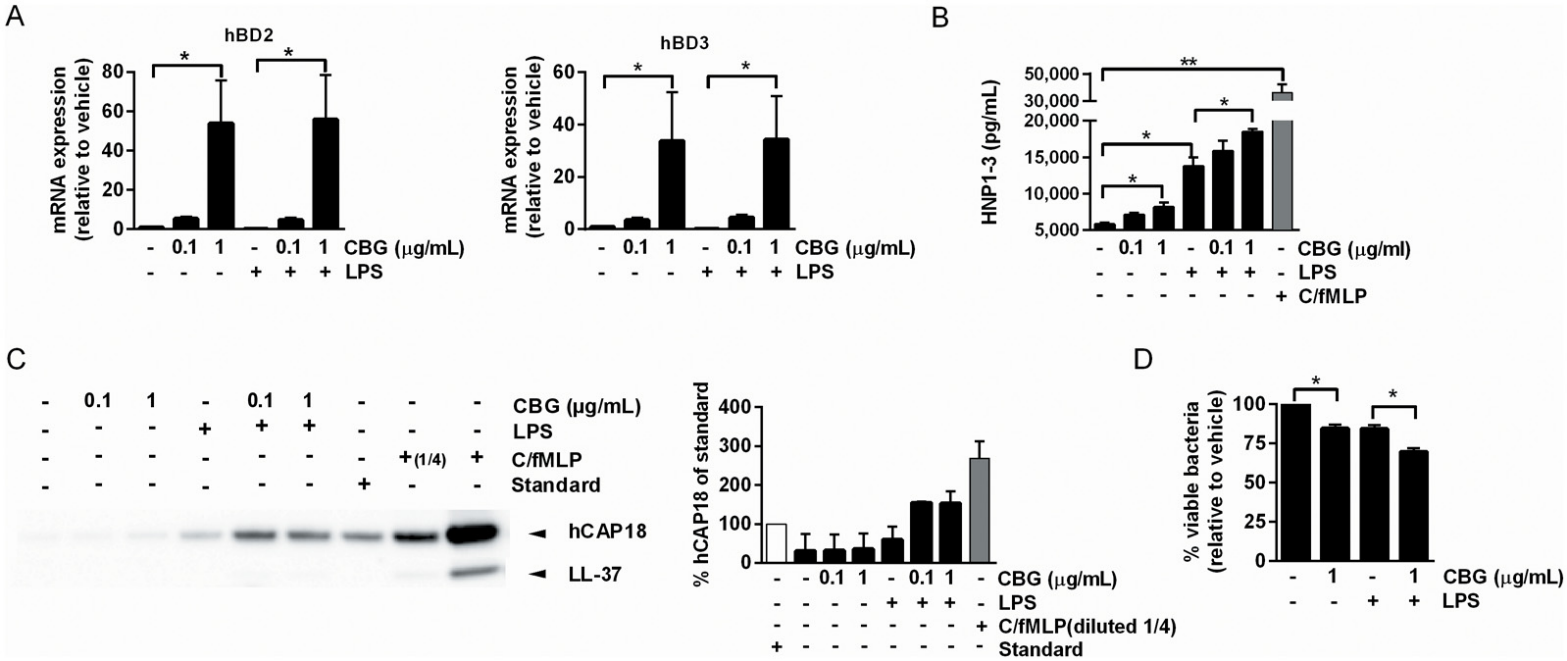
Induction of AMPs in DCs and neutrophils. (A) DCs were stimulated with vehicle or CBG in the absence or presence of LPS for 24 hours. Quantitative polymerase chain reaction (qPCR) for hBD-2 and hBD-3, normalized to GAPDH, was performed, and data shown represent mean gene expression (fold change compared to vehicle) + SEM for 3 donors. (B-D) Neutrophils were stimulated with vehicle or CBG in the absence or presence of LPS for 6 hours. (B) Neutrophil supernatants analyzed for release of HNP1-3 by ELISA. Cytochalasin B and fMLP (c/fMLP) treated cells served as positive control. Data shown represent means + SEM for 3 donors. *P<0.05. **P<0.01. (C) Neutrophil supernatants analyzed for release of proform hCAP-18 by semi-quantitative Western blot. Cytochalasin B/ fMLP (c/fMLP) served as positive control. Human serum containing 1 μg/ml hCAP-18 was used as a reference for relative determination of supernatant hCAP-18 levels. One representative donor out of two is shown (left) and data represent means + SD for 2 donors (right). (D) Bacterial killing was measured following incubation of neutrophils with live *S*. *pneumoniae* (strain T4R) at an MOI of 0.1 for 1 hour. Percentage live bacteria was calculated based on colony forming units (CFU) per ml obtained relative to vehicle-treated neutrophils. Data shown represent mean + SEM for 3 donors. *P<0.05.

In order to investigate possible immunomodulatory properties of CBG via the induction of AMPs, we measured the expression of the inducible β-defensins hBD-2 and hBD-3 in DCs exposed to CBG and found that mRNA levels were strongly upregulated (Fig 5A). However, protein levels of hBD-2 and hBD-3 were only slightly increased in DCs stimulated with CBG in the presence, but not absence, of LPS (S4 Fig) indicating that additional signal/s would be needed to ensure translation of these CBG-induced transcripts.

Neutrophils are instrumental in host antibacterial defense, and harbor antibacterial proteins and peptides which are stored in their granules and released upon activation through degranulation, a process in which the granules fuse with the cell membrane and release their content to the cell exterior. Since these cells are immediately recruited following an infectious assault, we assessed the effect of CBG on neutrophil granule content exocytosis and neutrophil survival. Interestingly, we observed that CBG induced release of the alpha-defensins HNP1-3, which are major constituents of neutrophil primary granules (Fig 5B). Importantly, the fraction of apoptotic and necrotic cells was not increased by CBG (S5 Fig). The second main human antimicrobial peptide belonging to the cathelicidin group is stored in neutrophil secondary granules as the proform hCAP-18. In contrast to HNP1-3, it is the proform hCAP-18 that is released from the granules, and the proteolytic processing to activate the antimicrobial peptide LL-37 occurs after degranulation. We therefore assessed the levels of the proform hCAP-18 in the cell supernatants by semi-quantitative Western blot, as a measure of hCAP-18/LL-37 release from neutrophil granules. As shown in Fig 5C, similar to the cytochalasin B and formyl-methionyl-leucyl-phenylalanine (c/fMLP) control, CBG triggered LPS-induced hCAP-18 exocytosis. Having shown that CBG induces release of AMPs from neutrophils, we next tested if it could induce bacterial killing by co-culturing live *S*. *pneumoniae* with CBG-treated neutrophils. Indeed there was a significant reduction in the number of viable bacteria upon incubation with CBG-treated neutrophils (Fig 5D). Together, these results indicate that besides modulating the innate immune response, CBG might mediate antibacterial effects at the site of infection by inducing antibacterial peptides.

## Discussion

Traditional Chinese medicines have been used for thousands of years to treat a variety of conditions in Asia, and they are also beginning to play a role in Western health care as complementary and alternative medicines. However, although they are widely used by the general population, their mechanisms of action are largely unknown. Chan-Su has long been used as a therapeutic agent to treat infections such as tonsillitis and acute pharyngitis; nevertheless, we could only detect a direct antimicrobial effect when Chan-Su was present at levels far above the levels documented following treatment with Chan-Su. Instead, we could show that CBG has potent immunomodulatory properties and may trigger anti-inflammatory and antibacterial effects at the site of infection. The observed down-regulated expression of DC maturation markers CD80, CD86 and MHC class II in LPS-stimulated cells suggests that CBG may favor the induction of tolerogenic DCs and thus inhibit their capacity to activate CD4^+^ T cells. The CBG-mediated dose-dependent suppression of DC cytokines in response to LPS, including IL-6, IL-8, IL-12 and IL-10, demonstrates that CBG has both pro- and anti-inflammatory effects, similar to what has been described for other cytokines such as TGF-β [35] but also compounds like antidepressants [36].

Despite the strong inhibition of multiple inflammatory cytokines, exposure of DCs to CBG resulted in a concurrent activation of caspase-1, leading to the release of active IL-1β. This pro-inflammatory cytokine is implicated in multiple immune reactions such as recruitment of inflammatory cells to the site of infection, and induction of AMPs, and it has been shown to contribute to resistance to bacterial infections [37–39]. Interestingly, stimulation with CBG alone activated caspase-1 to the same extent as co-stimulation with both CBG and LPS. However, stimulation with CBG alone resulted in lower levels of IL-1β in the supernatants. This may be explained by the difference in gene expression, since co-stimulation with CBG and LPS induced a 29-fold higher mRNA expression of IL-1β compared to stimulation with CBG alone (S6 Fig). Moreover, it has been shown that two functionally distinct inflammasome complexes can be found in pathogen-infected cells. The Asc/Caspase-1 complex which contains cleaved caspase-1 and is required for processing of IL-1β, and the Asc-independent inflammasomes with unprocessed caspase-1 that can induce cell death (so called “death-complexes”). The unprocessed caspase-1 in the death-complexes is active and can be detected by FLICA staining [40]. It is possible that CBG alone induces the formation of these death-complexes. Therefore caspase-1 is active, leading to cell death, but not to cleavage of pro-caspase-1 or IL-1β secretion. LPS is known to induce the production of the Asc-dependent NLRP3 inflammasome and pro-IL-1β, which could be the reason for the detection of cleaved caspase-1, as well as IL-1β secretion. Effects on capase-1 by CBG indicate that this compound has the ability to augment DC-mediated innate immunity against microorganisms.

Defensins are widely distributed in phagocytes and epithelial cells [41] and have been shown to play an important role in host defense to infection [42–45]. The β-defensins hBD-2 and hBD-3 are upregulated in human DCs in response to bacterial stimulation and may be important in the regulation of the innate immune response to pathogens in the oral cavity [46]. Furthermore, α-defensins HNP1-3 in human neutrophils have been shown to exhibit high antibacterial activity against pathogens such a *S*. *pneumoniae* [47]. We found that exposure of DCs to CBG upregulated mRNA expression, but not protein levels, of hBD-2 and hBD-3. However, release of α-defensins HNP1-3 and cathelicidin hCAP-18/LL-37 through neutrophil degranulation, was significantly increased, The exact mechanisms by which neutrophil stimulation leads to exocytosis remain poorly defined although intracellular calcium levels, small GTPases as well as MAPK kinas pathways have been implicated [48]. Future work will have to delineate if CBG functions via any of these mechanisms.

CBG belongs to a large family of cardiac glycosides that are known to exert their action by binding to the preassembled Na^+^/K^+^-ATPase signalosome, thereby activating a number of multiple signal transduction cascades including events ultimately leading to the activation of the mitogen-activated protein kinase cascade and to Ca^++^ oscillations [49]. It is likely that the immunomodulatory effects of CBG studied here is a result of such activation cascades; that however remains to be shown.

In conclusion, we show that CBG appears to regulate immune homeostasis in response to LPS stimulation by dampening DC maturation and cytokine/chemokine expression, while triggering caspase-1 mediated activation of IL-1β and enhancing host cell antimicrobial activities. Our findings give new insight into the mechanism of action of CBG in response to infection and provide evidence for the clinical application of Chan-Su as an anti-infective agent.

## Materials and Methods

### Reagents

CBG, with purity greater than 99%, was purchased from the Chinese National Institute for Food and Drug Control. It was dissolved in dimethyl sulfoxide (DMSO) from Sigma and stored in stock concentration of 20 mg/ml at -20°C. The Pierce Limulus Amebocyte Lysate (LAL) Chromogenic Endotoxin Quantitation Kit was purchased from Thermo Scientific. As a reference for mass spectrometry analysis showed in S1 Fig, CBG (C1272) was bought from Sigma-Aldrich, Sweden, dissolved to 1 mg/ml in MeOH. LPS from *Escherichia coli* O111:B4, adenosine 5′-triphosphate (ATP), staurosporine solution, cytochalasin B (CytB) and N-formyl-methionyl-leucyl-phenylalanine (fMLP) were purchased from Sigma. The caspase-1 inhibitor Z-YVAD-FMK was from MBL. The lactate dehydrogenase (LDH) Cytotoxicity Detection Kit ^PLUS^ was from Roche Applied Science.

### Mass spectrometry analysis of CBG

For mass spectrometry analysis, the CBG preparations were dissolved in MeOH. 10 μl sample in 50%MeOH/1%Formic acid final concentration was loaded per Spray Needle (Thermo Scientific) and electrosprayed offline to an LTQ-Orbitrap Velos Pro ETD mass spectrometer (Thermo Finnigan) using a spray voltage of 1.8 kV in the m/z range 100–1200. The resolution was 60 000 in the Fourier Transform analyzer (Orbitrap). Spectra were collected for 2–3 minutes. The mass spectrometry analysis was carried out at the Science for Life Laboratory, Mass Spectrometry Technology Platform in Uppsala, Sweden. The CBG preparation from China and the reference CBG preparation purchased from Sigma-Aldrich disclosed similar mass spectra (S1 Fig). The chemical formula of CBG is C28H34O6 (mass 442.24 Da) and C28H35O6 after protonation in the mass spectrometer (mass 443.24 Da). The 443.24 ion (CBG) dominated the spectrum. Also the 521.26, 885.48 and 907.46 masses were detected in both CBG preparations as well as sodium adducts (+22) of these ions. The mass 885.48 corresponds to (2*442.24+1), i.e. the protonated dimer of cinobufagin, and 907 is (2*442.24+23) the sodiated dimer of cinobufagin. The vehicle sample (DMSO) displayed no prominent masses above 371.10. This mass corresponds to siloxane and stems from the emitter of the mass spectrometer.

### Chan-Su extraction

The dried crude materials of Chan-Su (from Beijing Centre Biology Co., Ltd) were ground to fine particles and 2 g of powder was dissolved in 20 ml of 60% acetonitrile (ACN) and 1% trifluoroacetic acid (TFA), mixed at 4°C, incubated overnight, and then centrifuged at 19.000 rpm for 20 minutes. Supernatants were carefully collected, divided into small aliquots, lyophilized, and stored at -80°C. The extracts were dissolved in DMSO for experiments.

### Limulus amebocyte lysate (LAL) test

Presence of endotoxin from Gram-negative bacteria in the CBG preparation was assessed using the commercially available Thermo Scientific Pierce LAL Chromogenic Endotoxin Quantitation Kit. Solutions from the kit with known amount of endotoxin were used as positive control.

### Bacterial strains

The serotype 4 *S*. *pneumoniae* strain T4 (TIGR4; ATCC BAA-334) [50] was used in this study, as well as its isogenic mutant deficient in capsule (T4R) [51]. A clinical isolate of *S*. *pyogenes* [52] and the *E*. *coli* strain MC4100 were also used.

### Antimicrobial assay

The *S*. *pneumoniae* strains T4 and T4R were grown to OD_620_ 0.5 in tryptic soy broth (TSB) at 37°C, washed in PBS, and adjusted to 10^4^ bacteria per milliliter in 90% PBS and 10% TSB. The *S*. *pyogenes* clinical isolate was grown to OD_590_ 0.5 in TSB with 1% yeast (TSB-Y) at 37°C with shaking at 180 rpm, washed in PBS, and adjusted to 10^4^ bacteria per milliliter in 90% PBS and 10% TSB-Y. The *E*. *coli* strain MC4100 was grown to OD_590_ 0.5 in LB medium at 37°C with shaking at 180 rpm, washed in PBS, and adjusted to 10^4^ bacteria per milliliter in 90% PBS and 10% LB. Bacterial suspensions were mixed with Chan-Su (final concentration 50–15.000 μg/ml) and incubated at 37° with shaking (*S*. *pyogenes* and *E*. *coli*) or without shaking (*S*. *pneumoniae*) for 30 minutes. To determine the number of colony forming units, serial dilutions were plated and colony counts were performed the following day.

### Preparation of monocyte-derived DCs

Buffy coats from healthy donors were obtained from the blood bank at the Karolinska University Hospital (Immunology/Transfusion Medicine Clinic, http://www.karolinska.se/for-vardgivare/kliniker-och-enheter-a-o/kliniker-och-enheter-a-o/karolinska-universitetslaboratoriet/klinisk-immunologitransfusionsmedicin/bestallning-av-blodkomponenter-for-fou/). The buffy coats were provided anonymously; hence informed consent was not required. Monocytes were isolated using RossetteSep^™^ human monocyte enrichment kit (StemCell Technologies) and Ficoll-Paque PLUS (GE healthcare) according to the manufacturer′s instructions. To obtain DCs, the cells were then cultivated in RPMI 1640 medium (+L-glutamine) supplemented with 10% heat inactivated fetal bovine serum (FBS) (from Invitrogen) in the presence of 50 ng/ml human recombinant granulocyte-macrophage colony-stimulating factor (GM-CSF) and 50 ng/ml human recombinant interleukin (IL)-4 (PeproTech). On day 3, cells were given fresh media and cytokines (ratio 1:1) and cultured until day 6. The DC phenotype was assessed by examination of CD11c and CD1a expression using allophycocyanin (APC) conjugated mouse anti-human CD11c and fluorescein isothiocyanate (FITC) conjugated mouse anti-human CD1a (BD Pharmingen) before use.

### Stimulation of DCs

DCs (1×10^5^) were stimulated with vehicle (0.05% DMSO) or CBG from the Chinese National Institute for Food and Drug Control (0.1 and 1 μg/ml) in the absence or presence of LPS (100 ng/ml) in 96-well plates. ATP (5 mM), and staurosporine (0.05 and 0.5 μM) were also used. Each treatment for each donor was done in triplicate wells. In some experiments, cells were pretreated with 10 μM caspase-1 inhibitor Z-YVAD-FMK (from MBL) for 1 hour. Stimulated cells were incubated at 37°C in a humidified atmosphere with 5% CO_2_. After 24 hours, cells and supernatants were analyzed for expression of surface markers, caspases, AMPs, cytokines, and viability.

### Cytotoxicity assay

Cell-free supernatants were collected and analyzed for the release of the enzyme lactate dehydrogenase (LDH) using the Cytotoxicity kit (Roche) according to manufacturer’s instructions.

### Flow cytometry analysis

DCs were stimulated as described above and at 24 hours, cells were harvested and stained with fluorescently-labeled antibodies to CD80, CD86 and MHC class II, (all from BD Biosciences), or Annexin V-FITC and propidium iodide (PI) using the Annexin V-FITC Apoptosis Detection Kit according to the manufacturer’s protocol (BD Pharmingen). Early apoptotic cells were defined as Annexin V^+^ and PI^-^, and necrotic cells were defined as Annexin V^+^ and PI^+^. For the measurement of caspase activity, the FAM-FLICA^™^ Poly Caspase Assay Kit was used and for the measurement of active caspase-1, the FAM-FLICA^™^ Caspase 1 Assay Kit (both from ImmunoChemistry Technologies) was used. Cells were analyzed by flow cytometry (Gallios^™^ flow cytometer, Beckman Coulter) and at least 5000 cells were counted.

### Western blot analysis

Levels of caspase-1 and 3 were measured by Western blotting. Briefly, cells were lysed with RIPA buffer containing 1× protease inhibitors (Roche) on ice for 15 minutes. Cell debris was removed by centrifugation. Samples were diluted with lysis buffer and approximately 30 μg was loaded on to a 10% Bis-Tris gel (Invitrogen). Proteins were transferred to polyvinylidene fluoride (PDVF) membrane and blocked with 5% skim milk powder in PBS containing 0.1% Tween-20. Caspase-1 was detected using goat anti-human caspase-1 (1:100) (Santa Cruz), and caspase-3 was detected using rabbit anti-human caspase-3 (1:1000) (Cell Signaling Technology). As a loading control, a mouse anti-β-actin antibody (1:200) was used (Santa Cruz). Anti-goat IgG (1:5000), anti-rabbit IgG (1:10000) or anti-mouse IgG (1:5000) conjugated to horseradish peroxidase (GE Healthcare) were used as secondary antibodies. Blots were developed with Amersham^™^ ECL Plus Western blotting detection system (GE Healthcare), using a ChemiDoc^™^ XRS+ (Bio-Rad Laboratories).

Release of hCAP-18 from neutrophils was measured by Western blotting as previously described [53]. Briefly, samples were mixed with NuPAGE- SDS Sample buffer (Invitrogen, Life Technologies Europe BV, Stockholm, Sweden) and separated on a 4–12% NuPAGE Bis-Tris Gels (Invitrogen) with NuPAGE MES SDS running buffer (Invitrogen) and blotted onto polyvinylidene difluoride filters (Invitrogen). The filters were blocked with 2% w/v non-fat dry milk. hCAP-18 was detected using a custom-designed rabbit anti-LL-37 antibody, and a horseradish peroxidase-conjugated secondary goat anti-rabbit antibody (Dako AB, Stockholm, Sweden). Bound antibody was detected by chemiluminescence using SuperSignal West Dura (Pierce Biotechnology, Nordic Biolabs AB, Täby, Sweden) and ChemiDoc^™^ XRS+ (Bio-Rad Laboratories). Human serum (Prod no H4522, lot nr 113K048, Sigma-Aldrich, Stockholm, Sweden) that contains 1 μg/ml hCAP-18 as previously established [53], was used as a reference for the relative determination of hCAP-18 levels.

### Cytokine, chemokine and AMP detection by enzyme-linked immunosorbent assay (ELISA)

Cell supernatants were assayed for IL-1β and IL-10 (Ready-SET-Go! ELISA kits from eBioscience), IL-12p40, IL-8, IL-6 and TNF-α (OptEIA ELISA set from BD Biosciences), IL-18 (ELISA kit from MBL), hBD2, hBD3 and CXCL10 (mini ELISA kit from PeproTech), and HNP1-3 (ELISA kit from Hycult biotech).

### RNA isolation, cDNA synthesis and real time quantitative polymerase chain reaction

Total cellular RNA was extracted from stimulated cells using RNeasy Kit (Qiagen). The concentration and purity of isolated RNA was determined spectrophotometrically with Nanodrop ND 2000. Complementary DNA (cDNA) was synthesized from isolated RNA using the High Capacity cDNA Reverse Transcription kit (Applied Biosystems). Real-time polymerase chain reaction (PCR) was performed using iTaq^™^ Universal SYBR^®^ Green Supermix (BioRad Laboratories). Real-time PCR reactions were performed with an activation step at 95°C for 30 seconds, followed by 40 cycles of 95°C for 5 seconds and 55°C for 30 seconds. Pre-designed primer mixes containing forward and reverse primer for the specific real-time PCR target were purchased from Qiagen (QuantiTect Primer Assay). The following primers were used: IL-1beta (IL-1β) (Hs_IL1B_1_SG), Human Beta Defensin 2 (hBD-2) (Hs_DEFB4A_3_SG), hBD-3 (Hs_DEFB103B_2_SG), and glyceraldehyde 3-phosphate dehydrogenase (GAPDH) (Hs_GAPDH_1_SG). Each primer pair was validated for specificity by performing melt curve analysis of the PCR product to ensure the absence of primer dimers and unspecific products. For each sample, the mRNA expression level was normalized to the level of GAPDH and relative expression was determined with the ΔΔCT method. Each PCR run included a no-template control. All samples were assayed in triplicates.

### Purification and stimulation of human neutrophils

Neutrophils were isolated from human peripheral blood from healthy donors, provided by the blood bank at the Karolinska University Hospital (Immunology/Transfusion Medicine Clinic, http://www.karolinska.se/for-vardgivare/kliniker-och-enheter-a-o/kliniker-och-enheter-a-o/karolinska-universitetslaboratoriet/klinisk-immunologitransfusionsmedicin/). Blood was provided anonymously; hence informed consent was not required. Blood was diluted and layered on to Biocoll (Biochrom AG) and subjected to density gradient centrifugation. After washing and ammonium chloride lysis of RBCs, neutrophils were enriched using the EasySep human neutrophil enrichment kit (StemCell Technologies) by negative selection according to the manufacturer’s instructions. To ensure the cell purity, freshly isolated primary neutrophils were stained for neutrophil specific markers, CD16 and CD66b and analyzed by flow cytometry. Neutrophils were defined as CD66b^+^CD16^+^. The purity of the neutrophils post-enrichment was ~ 99%. Immediately after purification, cells (5×10^5^ per well) were suspended in RPMI 1640 medium supplemented with 2% heat inactivated FBS (from Invitrogen), treated with vehicle or CBG from the Chinese National Institute for Food and Drug Control (0.1 and 1 μg/ml) in the absence or presence of LPS (100 ng/ml) in 96-well plates and then cultured at 37°C, 5% CO_2_ atmosphere for up to 6 hours. To degranulate neutrophils, cells were first incubated with cytochalasin B (cytB) (5 μg/ml; Sigma-Aldrich) for 5 minutes at 37°C, followed by incubation with fMLP (10^−7^ M; Sigma-Aldrich) for a further 30 minutes [54]. After 6 hours, cells were harvested and stained with Annexin V-FITC (BD Bioscience) and fixable viability dye (FVD) (eBioscience) according to the manufacturer’s protocol, and then analyzed by flow cytometry (Gallios^™^ flow cytometer, Beckman Coulter). At least 10,000 cells were counted. Supernatants were collected for analysis of release of LDH, HNP1-3 and hCAP18/LL37.

### Neutrophil killing assay

Neutrophils (0.5x10^6^) were stimulated with CBG (1 μg/ml) in the absence or presence of LPS (100 ng/ml) for 6 hours. Cultures of *S*. *pneumoniae* (strain T4R) were grown to mid-log phase and added to neutrophils at an MOI of 0.1 in 96-well plates. The plates were then incubated at 37°C, and after 1 hour, reactions were stopped on ice and viable bacteria were quantified by serial plating on blood agar plates. Percentage bacterial viability was calculated based on the ratio of number viable colony forming units (CFU) per ml obtained relative to vehicle treated neutrophils.

### Statistical analysis

Statistical analysis was performed with GraphPad Prism 5.0 software. Differences were tested for significance using ANOVA and *p*<0.05 was considered statistically significant. Results shown represent means + standard errors of the mean (SEM) of at least 3 donors, unless otherwise specified.

## Supporting Information

S1 Fig Mass spectrometry analysis of CBG preparation and vehicle (DMSO). (A) CBG (China), (B) CBG reference (Sigma), (C) vehicle (DMSO). The mass corresponding to CBG (m/z) 443.24 is marked with an arrow. The relative intensities of the peaks cannot be used for estimation of the amount of impurities since different compounds have different ionization efficiencies. However the relative amount between the samples can be compared.
(TIF)

S2 Fig No endotoxins (LPS) were detected in CBG solution. Presence of endotoxin was assessed through a chromogenic LAL test, using Thermo Scientific Pierce Limulus Amebocyte Lysate (LAL) Chromogenic Endotoxin Quantitation Kit. Solutions with known amounts of endotoxins units (EU) were used as positive control.
(TIF)

S3 Fig CBG alone does not affect DC cytokine production. DCs were stimulated with vehicle or CBG for 24 hours. Supernatants were collected and analyzed for IL-8, IL-12p40, IL-10 and TNF-α. Data shown represent means + SEM of cytokine production for 3 donors.
(TIF)

S4 Fig CBG increases hBD-2 and hBD-3 expression in the presence of LPS in DCs. DCs were stimulated with vehicle or CBG in the absence or presence of LPS (100 ng/ml) for 24 hours. Supernatants were collected and analyzed for hBD-2 (A) and hBD-3 (B). Data shown represent means + SEM of protein release for 3 donors.
(TIF)

S5 Fig CBG does not affect cell viability of neutrophils. Neutrophils were stimulated with vehicle or CBG in the absence or presence of LPS (100 ng/ml) for 6 hours. Cell apoptosis and necrosis was examined by analyzing the percentage of Annexin V^+^ or Annexin V^+^ and PI^+^ cells, respectively. Data shown represent means + standard deviation (SD) for 1 donor, and are representative of the results of 3 donors.
(TIF)

S6 Fig CBG upregulates mRNA expression of IL-1β in DCs. DCs were stimulated with vehicle or CBG in the absence or presence of LPS (100 ng/ml) for 6 hours. Quantitative polymerase chain reaction (qPCR) for IL-1β, normalized to GAPDH, was performed, and data shown represent mean gene expression (fold change compared to vehicle) + SEM for 4 donors.
(TIF)
