# Supplementary figures and images for: Cinobufagin Modulates Human Innate Immune Responses and Triggers Antibacterial Activity
Source: PLoS One. 2016 Aug 16;11(8):e0160734. doi: 10.1371/journal.pone.0160734 (PMC4986986; doi:10.1371/journal.pone.0160734)

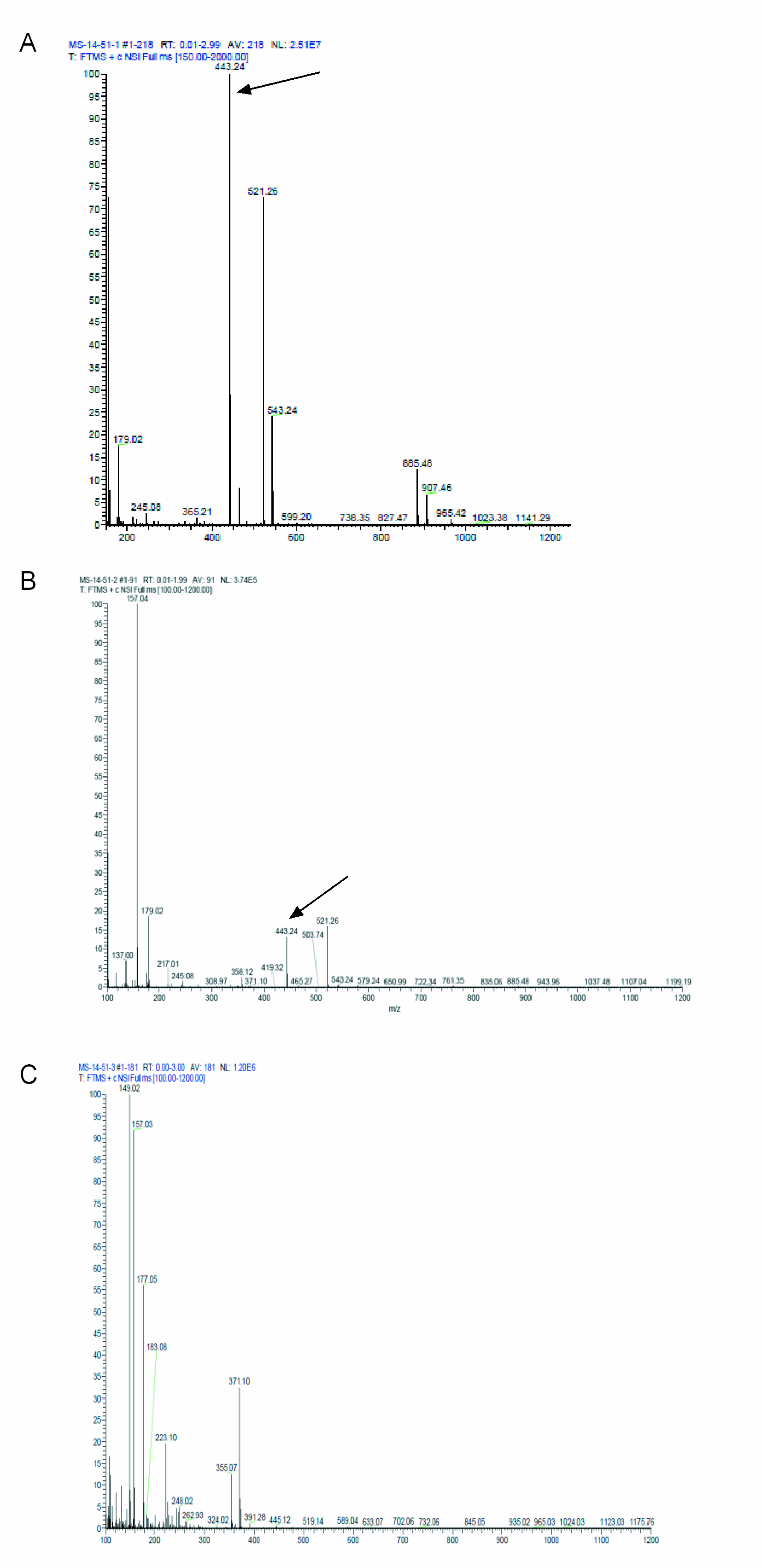

Supplement: S1 Fig — (A) CBG (China), (B) CBG reference (Sigma), (C) vehicle (DMSO). The mass corresponding to CBG (m/z) 443.24 is marked with an arrow. The relative intensities of the peaks cannot be used for estimation of the amount of impurities since different compounds have different ionization efficiencies. However the relative amount between the samples can be compared. (TIF) [file pone.0160734.s001.tif]

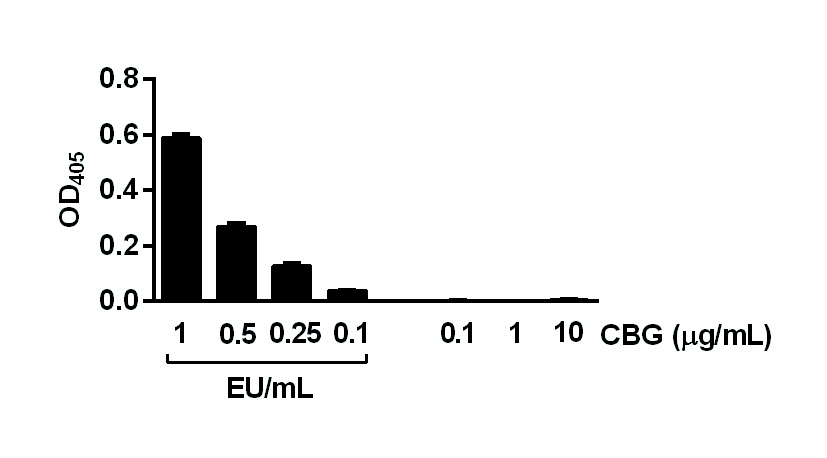

Supplement: S2 Fig — Presence of endotoxin was assessed through a chromogenic LAL test, using Thermo Scientific Pierce Limulus Amebocyte Lysate (LAL) Chromogenic Endotoxin Quantitation Kit. Solutions with known amounts of endotoxins units (EU) were used as positive control. (TIF) [file pone.0160734.s002.tif]

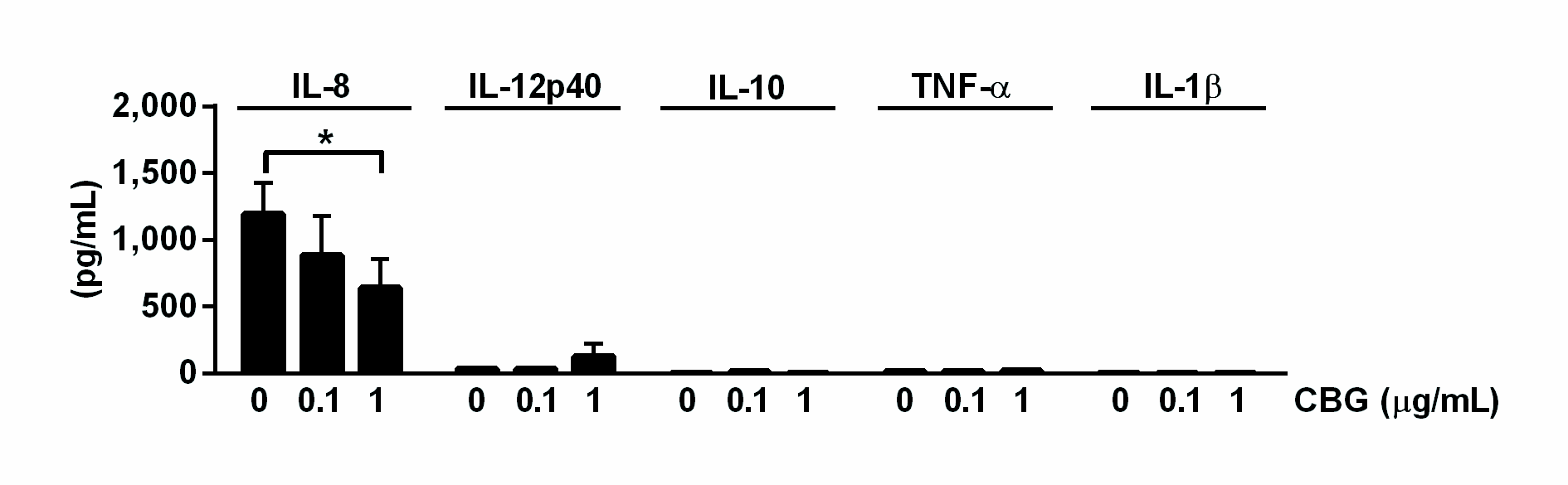

Supplement: S3 Fig — DCs were stimulated with vehicle or CBG for 24 hours. Supernatants were collected and analyzed for IL-8, IL-12p40, IL-10 and TNF-α. Data shown represent means + SEM of cytokine production for 3 donors. (TIF) [file pone.0160734.s003.tif]

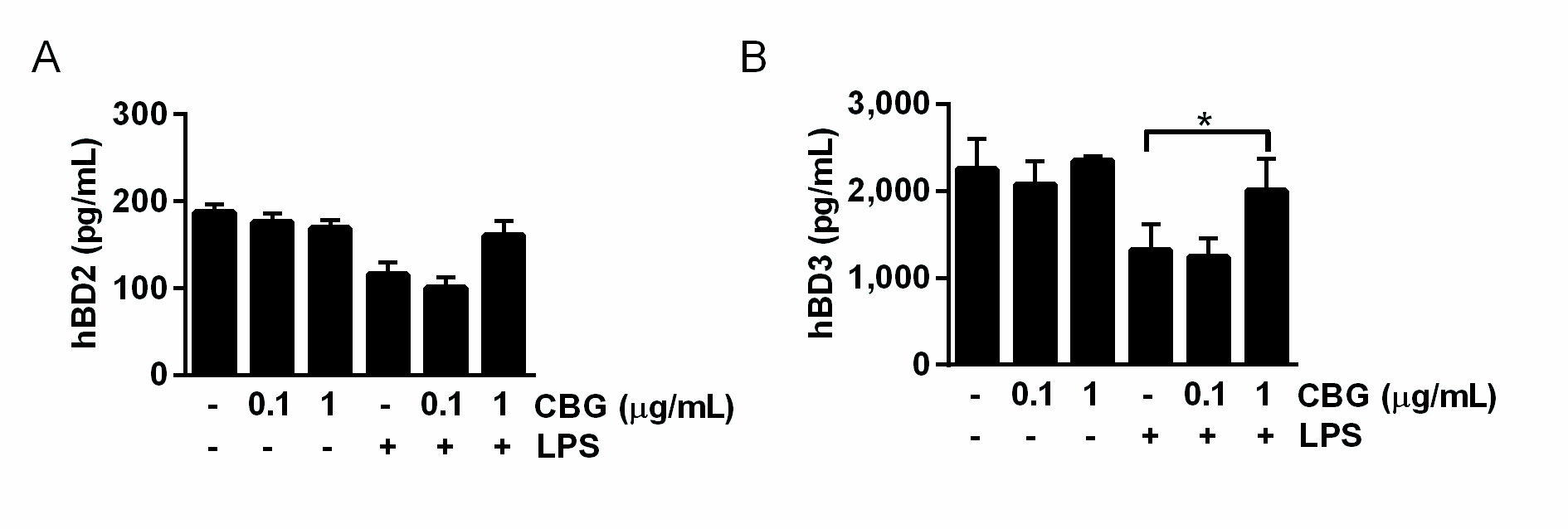

Supplement: S4 Fig — DCs were stimulated with vehicle or CBG in the absence or presence of LPS (100 ng/ml) for 24 hours. Supernatants were collected and analyzed for hBD-2 (A) and hBD-3 (B). Data shown represent means + SEM of protein release for 3 donors. (TIF) [file pone.0160734.s004.tif]

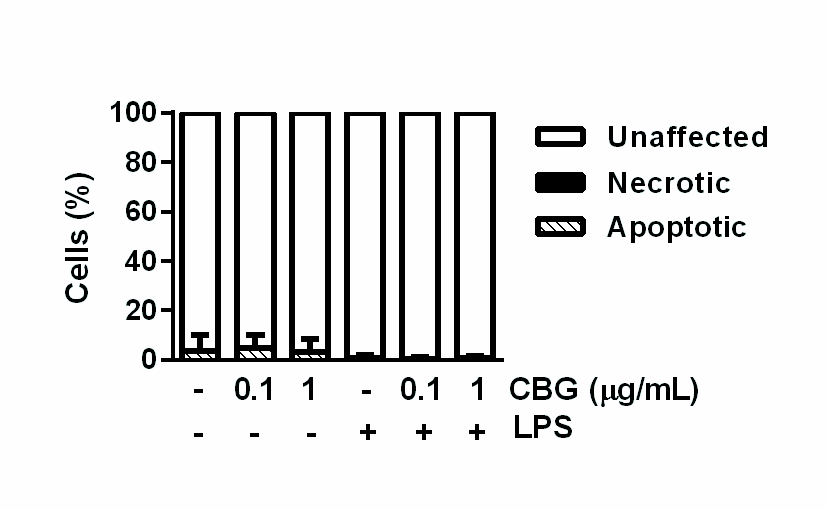

Supplement: S5 Fig — Neutrophils were stimulated with vehicle or CBG in the absence or presence of LPS (100 ng/ml) for 6 hours. Cell apoptosis and necrosis was examined by analyzing the percentage of Annexin V+ or Annexin V+ and PI+ cells, respectively. Data shown represent means + standard deviation (SD) for 1 donor, and are representative of the results of 3 donors. (TIF) [file pone.0160734.s005.tif]

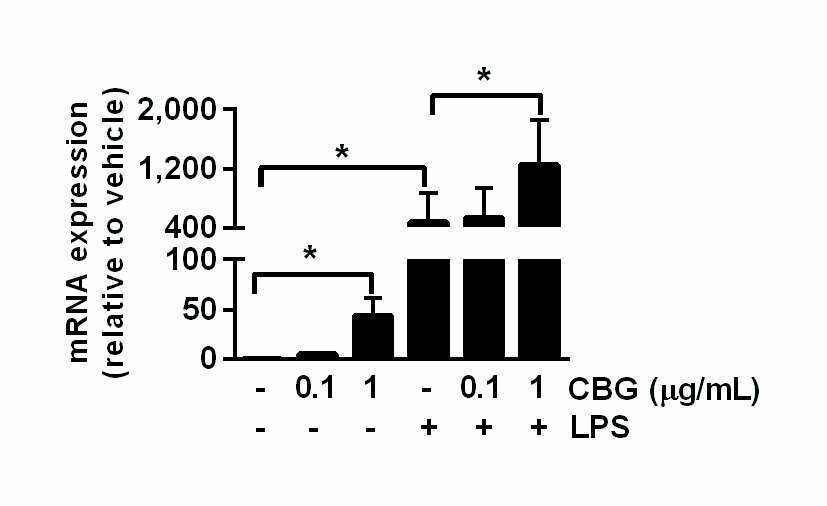

Supplement: S6 Fig — DCs were stimulated with vehicle or CBG in the absence or presence of LPS (100 ng/ml) for 6 hours. Quantitative polymerase chain reaction (qPCR) for IL-1β, normalized to GAPDH, was performed, and data shown represent mean gene expression (fold change compared to vehicle) + SEM for 4 donors. (TIF) [file pone.0160734.s006.tif]
